# Greater aortic stiffness is associated with renal dysfunction in participants of the ELSA-Brasil cohort with and without hypertension and diabetes

**DOI:** 10.1371/journal.pone.0210522

**Published:** 2019-02-04

**Authors:** Júlia S. A. Cândido, Lidyane V. Camelo, José Geraldo Mill, Paulo A. Lotufo, Antonio Luiz P. Ribeiro, Bruce B. Duncan, Luisa C. C. Brant, Sandhi Maria Barreto

**Affiliations:** 1 Medical School & Clinical Hospital, Universidade Federal de Minas Gerais, Belo Horizonte, Minas Gerais, Brazil; 2 Department of Physiological Sciences, Universidade Federal do Espírito Santo, Vitória, Espirito Santo, Brazil; 3 Center for Clinical and Epidemiologic Research, Universidade de São Paulo, São Paulo, Brazil; 4 Medical School, Universidade Federal do Rio Grande do Sul, Porto Alegre, Rio Grande do Sul, Brazil; Shanghai Institute of Hypertension, CHINA

## Abstract

**Background:**

Arterial stiffness has been associated with renal dysfunction and its progression, but the pathophysiological relation underlying this association has not been fully established, particularly among individuals without hypertension and diabetes. We investigated the cross-sectional associations between arterial stiffness and renal function in adults without cardiovascular disease, and whether this association remained among subjects without hypertension and diabetes.

**Methods:**

All eligible participants from ELSA-Brasil (2008–2010), aged 35 to 74 years (N = 13,586) were included, of whom 7,979 were free from hypertension and diabetes. The response variables were: 1) low glomerular filtration rate (eGFR<60ml/min/1.73m^2^) estimated by CKD-EPI; 2) increased albumin/creatinine ratio (ACR ≥30mg/g); and 3) chronic kidney disease (CKD). Arterial stiffness was ascertained by the carotid-femoral pulse wave velocity (PWV). The covariates were sex, age, race/color, level of schooling, smoking, body mass index, total cholesterol/HDL-c glycated hemoglobin, diabetes, systolic blood pressure, heart rate and use of antihypertensive drugs. Logistic regression was used to examine the associations.

**Results:**

After all adjustments, 1 m/s increase in PWV was associated with ORs equal to 1.10 (95%CI: 1.04–1.16), 1.10 (95%CI: 1.05–1.16) and 1.12 (95%CI: 1.08–1.17) of low eGFR, high ACR, and CKD, respectively. In subjects without hypertension and diabetes, these ORs were 1.19 (95%CI: 1.07–1.33), 1.20 (95%CI: 1.07–1.32) and 1.21 (95%CI: 1.11–1.30), respectively.

**Conclusion:**

The increase in PWV was associated with all renal dysfunction markers, even in individuals without hypertension and diabetes, suggesting a relation that is not completely mediated by the presence of these conditions.

## Introduction

Arterial stiffness results from a structural change in the arteries related to excess production of collagen fibers and/or abnormal loss of elastin. It is an established risk factor for cardiovascular disease (CVD) and has also been associated with the incidence and progression of chronic kidney disease (CKD) [1,2] and cardiovascular mortality in patients with CKD [3,4]. The kidneys have a low impedance and resistance vascular system; thus, they are passively perfused by high flow and prone to greater transmission of pulsatile energy and glomerular injury, as a consequence of increased arterial stiffness [5–7].

In addition, increased arterial stiffness leads to greater pressure variability and contributes to functional changes in the cardiovascular system, including the microcirculation of organs, such as kidneys and brain [1,8–10]. When arterial stiffness increases, vascular networks of the renal system are exposed to greater pressure and pulsation oscillations [8,11], since the kidneys, unlike other organs, are easily affected by increases in blood pressure [1,12], which can potentially damage glomerular capillaries. Therefore, it is suggested that increased arterial stiffness plays a role in the early stages of renal dysfunction [13].

The positive association between arterial stiffness and renal dysfunction has been reported mainly among subjects with end-stage CKD [14–16] but has also been found in subjects at early stages of CKD [17–21] and in population-based studies [3,18,22]. Similarly, clinical trials have also shown a positive association between arterial stiffness and disease progression, including the initiation of replacement therapy in individuals at advanced stages of CKD [16,19,20,23].

Although this possible pathophysiological explanation on why greater arterial stiffness is associated with renal dysfunction has already been shown in previous studies, there are still inconsistencies in the literature [7,13]. Especially, we do not know whether the relation between arterial stiffness and CKD is fully explained by the presence of hypertension (hypertension) and diabetes (diabetes), the main risk factors for the onset of CKD [24–26] and CVD. The only population-based study so far that examined the association between pulse wave velocity (PWV) and albumin/creatinine ratio (ACR) in subjects with and without hypertension or diabetes found a stronger association among individuals with these conditions as compared to those without them [18]. However, the above-mentioned study did not examine the association between PWV and estimated glomerular filtration rate (eGFR) nor did it verify whether the association remained significant among subjects without both hypertension and diabetes simultaneously.

This study aims to help closing these gaps related to investigating the association between carotid-femoral PWV and renal dysfunction as measured by eGFR, ACR and CKD in middle-aged adults, with no cardiovascular disease, and in a subset of participants from the same cohort free of hypertension and diabetes. Our hypothesis is that arterial stiffness is associated with renal dysfunction as measured by the three variables, both in the general population and in the subgroup without hypertension and diabetes.

## Materials and methods

This study used baseline data from the ELSA-Brasil (2008–2010), a multicenter cohort study of 15,105 civil servants, aged 35 to 74 years, active and retired, recruited from higher education and research institutions from six Brazilian cities: Universidade Federal de Minas Gerais (UFMG)—Belo Horizonte, Univesidade Federal do Rio Grande do Sul (UFRGS)—Porto Alegre, Fundação Oswaldo Cruz (Fiocruz)—Rio de Janeiro, Universidade Federal da Bahia (UFBA)—Salvador, Universidade de São Paulo—São Paulo and Universidade Federal do Espírito Santo (UFES)—Vitória. The main objectives of ELSA-Brasil were to investigate the incidence and progression of diabetes and CVD; and examine the biological, behavioral, environmental, occupational, psychological and social factors related to these diseases and their complications, trying to build a causal model that reflects their interrelations [27]. During baseline, all participants were submitted to face-to-face interviews, clinical examinations, anthropometric measurements, and laboratory and imaging examinations conducted by trained and certified research assistants [27]. More details on the study methodology and the cohort profile have been previously described [27–29].

The ELSA-Brasil was approved by the National Committee for Ethics in Research (CONEP Brazil, no. 976/2006), and by each of the following ethics review boards of the sites involved in the study: UFMG Research Ethics Committee; UFRGS Research Ethics Committee; Fiocruz Research Ethics Committee; UFBA Committee for Ethics in Research with Human Beings of the Health Sciences Institute; USP Code of Ethics; and UFES Human Research Ethics Committee.

### Population of the present study

Out of 15,105 participants of ELSA-Brasil, those with unvalid PWV data (N = 380), missing data for serum creatinine (N = 6) and ACR (N = 426), or with self-reported CVD (N = 707) were excluded. Thus, the total sample of this study was 13,586 subjects.

### Study variables

#### Evaluation of renal function

The response variables were eGFR, ACR and CKD. The participants were submitted to a 12-hour urine volume test and blood tests after a 12-hour fast [28]. Creatinine was measured by the colorimetric enzymatic assay (Jaffé method) and microalbuminuria was determined by immunochemical assay (nephelometry) [27]. Subjects were classified according to the ACR into risk categories under 30 mg/g and greater than or equal to 30 mg/g.

CKD-EPI equation was used to estimate the eGFR (eGFR) with no correction for race/color [30]. Subjects were classified according to the eGFR into risk categories < 60 ml/min/1.73 m^2^ or ≥60 ml/min/1.73 m^2^. CKD was defined as values eGFR <60 ml/min/1.73 m^2^ or ACR ≥ 30 mg/g.

#### Evaluation of arterial stiffness

Arterial stiffness was measured by the PWV obtained by a validated automatic device (Complior, Artech Medicale, France), with the subject lying in a room with a temperature between 20°C and 24°C [31,32]. The PWV measures aortic stiffness, which is the main parameter reflecting the buffering properties of the arterial bed [33], and is an independent predictor of cardiovascular events in different populations [2,34]. Before the PWV measurement, blood pressure was measured with the subject lying down using an oscillometric device (Omron HRM 705 CP) on the right arm. Measurement of the distance from the suprasternal notch to the right femoral pulse was performed with a tape measure. Pulse sensors were positioned in the right carotid and femoral arteries, allowing the visualization of pulse waves on a computer screen [31].

A software identifies the pulse waves with good recording quality. The PWV is calculated by dividing the distance from the suprasternal notch to the femoral pulse by the time lag between the carotid and femoral pulses. The PWV of each participant was calculated by the arithmetic mean obtained in ten consecutive cardiac cycles at a regular heart rate [31].

#### Covariables

The covariables included age, sex, race/color (black, white, brown (“pardo”—mixed), Asian, Brazilian indigenous), level of schooling (incomplete middle school, middle school, complete high school, undergraduate studies), smoking (Yes—smokers/No—nonsmokers or former smokers), total cholesterol/HDL-C ratio, glycated hemoglobin, body mass index (BMI), diabetes, systolic blood pressure (SBP), heart rate (HR) and use of antihypertensive drugs. Alcohol consumption was assessed via questionnaire and was dichotomized according to the amount ingested per week (men ≥ 210 g; women ≥ 140 g). Level of Total cholesterol and HDL-c were measured using standardized automated enzymatic colorimetric methods on blood samples collected after a 12-hour fast. Glycated hemoglobin was measured using HPLC (Bio-Rad D-10 Dual Program Laboratories. BMI was defined as weight divided by squared height and classified according to the standard definition. The presence of diabetes was defined by a reported medical diagnosis of diabetes and/or use of medication for diabetes and/or fasting glycemia greater or equal 126 mg/dL, and/or 75 g oral glucose tolerance test greater or equal 200 mg/dL, and/or Hb A1C greater or equal 6.5%. Blood pressure was measured immediately before the PWV measurement, with the subject lying down, using an oscillometric device (Omron HRM 705 CP) on the right arm [31]. Information on the use of antihypertensive medications was obtained based on subjects' self-reports. Hypertension was defined by systolic blood pressure ≥ 140mmHg and/or diastolic blood pressure ≥ 90 mmHg and/or use of antihypertensive medication. HR was measured three times after a 5-minute rest, with the subject sitting up, using a validated oscilometric device (Omron HEM-705 CP), and then it was estimated based on the arithmetic mean of the second and third measurements.

### Data analysis

Logistic regression models were used to evaluate the association between the PWV and the three endpoints of interest: low eGFR, increased ACR and presence of CKD. Firstly, a univariate analysis was performed between the PWV and each renal function variable (Model 0). Model 1 included adjustment by age, sex, race/color, and schooling. Model 2 added to model 1 the variables smoking, BMI, glycated hemoglobin, and total cholesterol/HDL ratio. Model 3 added the variables diabetes, use of antihypertensive medications, SBP and HR to model 2. In the final model having low eGFR as an endpoint included adjustment by ACR (continuous), and the model for increased ACR included adjustment by GFR (continuous). Alcohol consumption was added to the models but it did not remain statistically significant, and, for this reason it was not retained in any of the final models.

Finally, the same analyses were performed considering only the participants without hypertension or diabetes. As a result, the adjustment variables diabetes and use of antihypertensive medications were excluded from model 3. The level of significance was set at 5%.

All the analyses were performed using the Stata 14.0 software (Stata Corporation, College Station, USA).

## Results

Among the 13586 participants of the ELSA-Brasil baseline enrolled in this study, the mean age was 51.7±9 years, 54.5% were female, and mostly self-reported their race/color as White (52.6%), and had complete higher education (53.3%), 95.8% of the subjects had eGFR higher than 60 ml/min/1.73m^2^ mean, 95.3% had ACR below 30 mg/g and 8.2% had CKD. The mean PWV was 9.3 ± 1.8m/s (Table 1). The means of eGFR (S1 Table), ACR (S2 Table), PWV (S3 Table) and prevalence of CKD (S4 Table) according to age groups and sex can be seen in the Supporting information.

**Table 1:** Descriptive characteristics of participants from baseline of the Brazilian longitudinal study of adult health (ELSA-Brasil), 2008–2010, (N = 13586).

Characteristics	% or mean (SD)	
	General population	Without hypertension and diabetes
	N: 13586	N: 8212
Age (years), mean (SD)	51.7 (9)	49.5 (8.4)
Sex, (%)		
*Female*	54.5	58.1
Race/color, (%)		
*White*	52.6	57.2
*Brown*	27.9	27.0
*Black*	15.9	12.3
*Asian descendent*	2.5	2.5
*Brazilian Indigenous*	1.0	0.9
Level of Schooling, (%)		
*Undergraduate studies*	53.3	59.2
*Complete high school*	34.7	32.5
*Middle school*	6.5	4.9
*Incomplete middle school*	5.4	3.4
Smoking, (%)	13.1	13.2
Total cholesterol/HDL-C ratio, mean (SD)	3.96 (1.0)	3.84 (0.1)
Glycated hemoglobin, mean (SD)	5.45 (0.9)	5.18 (0.5)
Body mass index, mean (SD)	26.8 (4.6)	25.8 (4.2)
Diabetes Mellitus, (%)	14.7	-
Hypertension, (%)	33,8	-
Use of antihypertensive, (%)	26.9	-
Heart rate (bpm), mean (SD)	70 (10)	69.9 (9.6)
Systolic blood pressure (mmHg), mean (SD)	126 (18)	119 (12)
eGRF*<60 ml/min/1*,*73m^2^*, (%)	4.2	2.0
Albumin/creatinine ratio *≥30 mg/g*, (%)	4.7	2.2
CKD, (%)	8.2	4.1
PWV (m/s) mean (SD)	9.3 (1.8)	8.7 (1.4)

**Fig 1:**
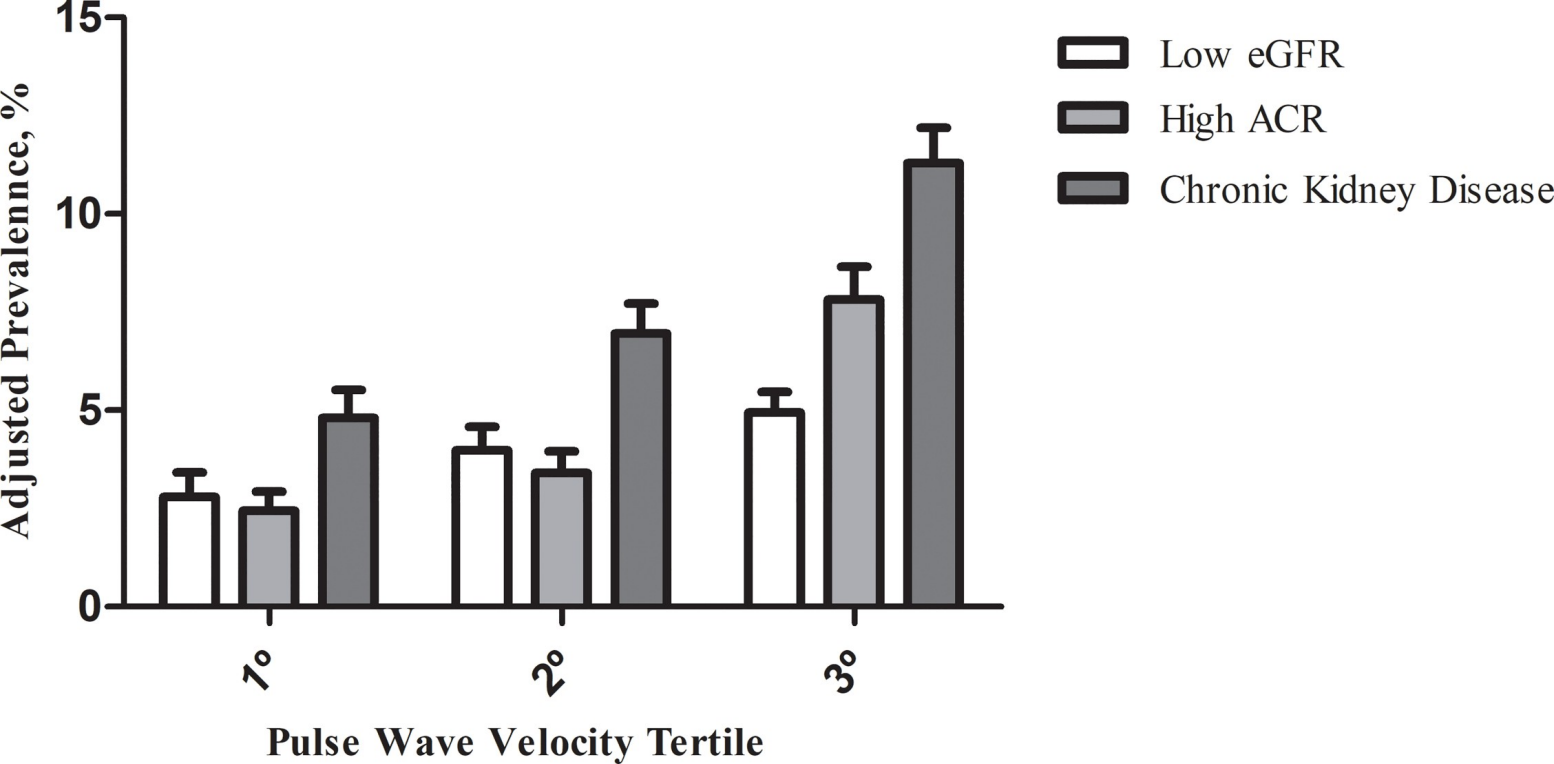
Prevalence of low estimated glomerular filtration rate (eGFR), elevated serum albumin/creatinine ratio (ACR), and chronic kidney disease according to tertile of pulse wave velocity adjusted by age, sex and race/color, and 95% confidence interval in the general population (ELSA-Brasil, 2008–2010).

**Fig 2:**
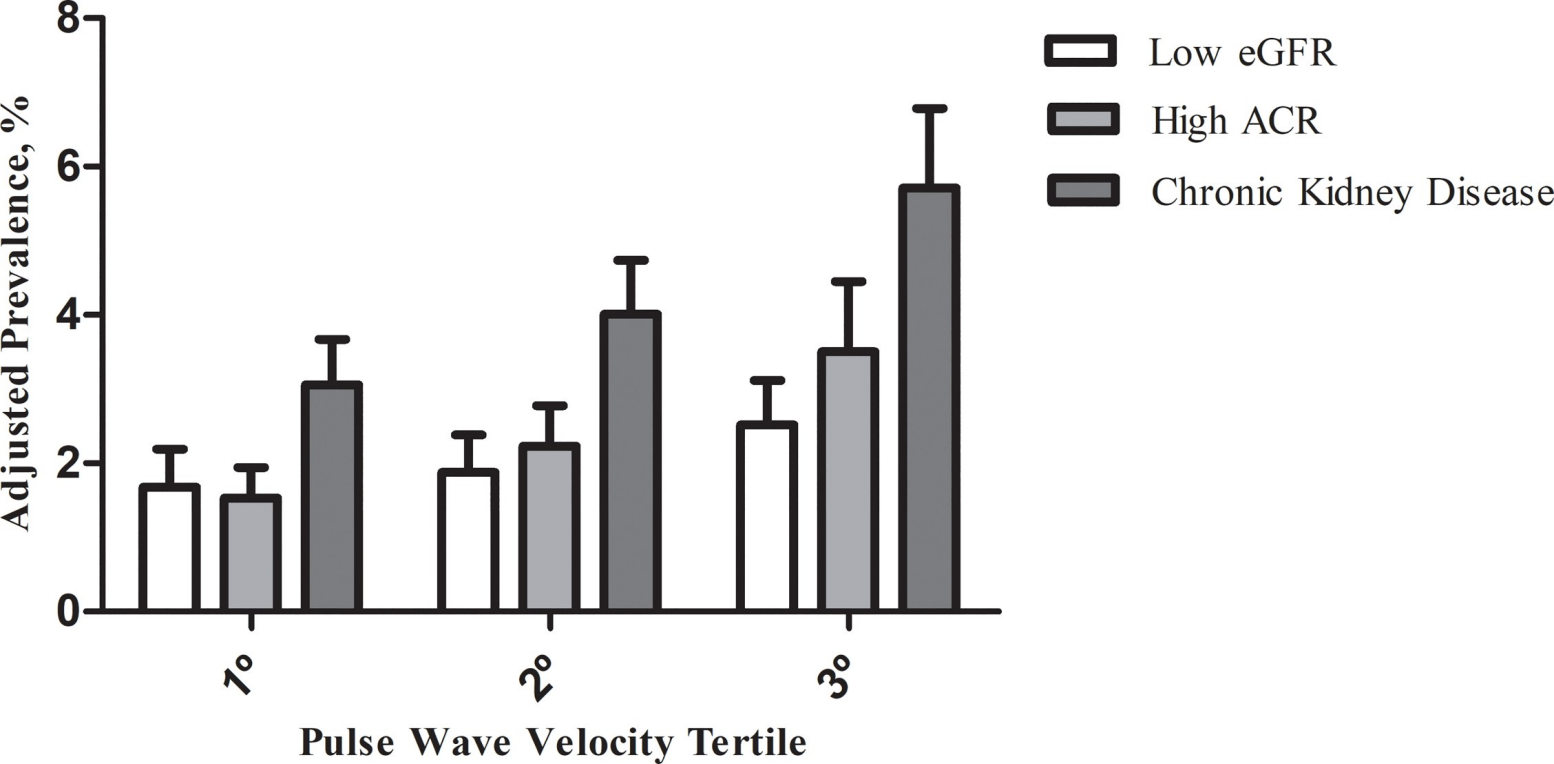
Prevalence of low estimated glomerular filtration rate (eGFR), elevated serum albumin/creatinine ratio (ACR), and chronic kidney disease according to tertile of pulse wave velocity adjusted by age, sex and race/color, and 95% confidence interval in adults free of hypertension and diabetes (ELSA-Brasil, 2008–2010).

When we investigated the prevalence of eGFR, ACR and CKD according to tertiles of PWV adjusted for age, sex- and race (Fig 1), we found that those in the highest PWV tertile had the highest prevalences of low eGFR, high ARC and CKD, and the figure also suggests an increasing gradient from the 1^st^ to the 3^rd^ PWV distribution tertile. After excluding subjects with hypertension and diabetes (Fig 2), the prevalences of low eGFR, high ARC and CKD are lower than those of the general population, but the same tendency of increasing gradient in the prevalence between the 1° and 3° tertiles of the PWV is noticed.

In the univariate analysis of the association between PWV and low eGFR, a 1m/s increase in PWV was associated with a 38% increase in the odds of GFR <60 ml/min/1.73m^2^. After adjusting for sociodemographic variables, there was a marked reduction in the magnitude of the association (OR: 1.13; 95%CI: 1.08–1.18), particularly when adding age as a variable. Behavioral and clinical factors led to a slight decrease in the magnitude of the association (OR: 1.11; 95%CI: 1.07–1.17), and after adding the ACR to the final model, each 1 m/s increase in the mean PWV led to a 10% increase in the odds of low eGFR (OR 1.10; 95%CI: 1.04–1.16) (Table 2).

**Table 2:** Logistic regression models of the association between pulse wave velocity (m/s) and renal function markers in adults without CVD, participants in the ELSA-Brasil cohort baseline, 2008–2010.

Models	eGFR < 60ml/min/1,73m^2^	ACR ≥30 mg/g	Chronic Kidney Disease
	OR (95% CI) ^1^	OR (95% CI) ^1^	OR (95% CI) ^1^
Univariate model	1.38 (1.33; 1.43)***	1.37 (1.33;1.42)***	1.40 (1.37;1.45)***
Model 1 –adjusted by age, sex, race/color and schooling	1.13 (1.08;1.18)***	1.31 (1.26;1.36)***	1.23 (1.19;1.27)***
Model 2 –model 1 + smoking, BMI, HbA1c, total cholesterol/HDL-C	1.11 (1.07; 1.17)***	1.25 (1.20;1.30)***	1.19 (1.15;1.23)***
Model 3—model 2 + diabetes, antihypertensive drugs, SBP e HR	1.11 (1.06;1.17)***	1.12 (1.06;1.17)***	1.12 (1.08;1.17)***
Model 4^2^ –model 3 + ACR or eGFR	1.10 (1.04;1.16)**	1.10 (1.05;1.16)***	___

^1^ The OR represent the chances of low eGFR and high ACR related with an increase of 1m/s in pulse wave velocity.

^2^Model 4 for GFR was adjusted by ACR and e Model 4 for ACR was adjusted by eGFR.

**p<0.01

***p<0.001 OR: odds ratio

CI: confidence interval. eGFR: glomerular filtration rate. ACR: albumin/creatinine ratio. BMI: Body mass index. HbA1c: glycated hemoglobin HR: heart rate. SBP: systolic blood pressure.

As for the relation between the PWV and elevated ACR, we observed a greater reduction in the magnitude of the association after adding behavioral and clinical factors to the initial models (Model 3), and this difference was observed particularly when adding pressure-related variables. The final adjustment for eGFR did not significantly alter the magnitude of the association between PWV and elevated ACR (OR: 1.10; 95%CI: 1.05–1.16; p<0.001 (Table 2). In conclusion, the results in Table 2 show, in the crude model of the association between PWV and CKD, that the odds of CKD increased by 40% with every 1 m/s increase in the mean PWV and, after all adjustments, the magnitude of the association increased to 12% (OR: 1.12; 95% CI: 1.08–1.17).

The results of the analysis restricted to subjects with no hypertension and/or diabetes (Table 3) were similar to those of the previous analysis, i.e., with statistically significant associations between PWV and low eGFR, elevated ACR and CKD. In the final models regarding the three response variables, the magnitudes of the associations, after all adjustments, are higher than those in Table 2. At every 1 m/s increase in the mean PWV, the odds of eGFR <60 ml/min/1.73m^2^, ACR ≥30 mg/g and CKD increased by 19%, 20% and 21%, respectively (Table 3).

**Table 3:** Logistic regression models of the association between pulse wave velocity (m/s) and renal function markers in adults without CVD and free of hypertension and diabetes, participants in the ELSA-Brasil cohort baseline, 2008–2010.

Models	eGFR < 60ml/min/1,73m^2^	ACR ≥30 mg/g	Chronic Kidney Disease
	OR (95% CI) ^1^	OR (95% CI) ^1^	OR (95% CI) ^1^
Univariate model	1.44 (1.33;1.55)***	1.27 (1.17;1.38)***	1.37 (1.28;1.45)***
Model 1 –adjusted by age, sex, race/color and schooling	1.16 (1.05;1.28)**	1.22 (1.11;1.34)***	1.21 (1.13;1.30)***
Model 2 –model 1 + smoking, BMI, HbA1c, total cholesterol/HDL-C	1.15 (1.05;1.27)**	1.22 (1.11;1.35)***	1.20 (1.12;1.29)***
Model 3—model 2 + SBP e HR	1.20 (1.08;1.33)**	1.19 (1.07;1.32)**	1.21 (1.11;1.30)***
Model 4^2^ –model 3 + ACR or eGFR	1.19 (1.07;1.33)**	1.20 (1.07;1.32)**	___

^1^ The OR represent the chances of low eGFR and high ACR related with an increase of 1m/s of pulse wave velocity.

^2^Model 4 for GFR was adjusted by ACR and e Model 4 for ACR was adjusted by eGFR

**p<0.01

***p<0.001.

OR: odds ratio, CI: confidence interval. eGFR: glomerular filtration rate. ACR: albumin/creatinine ratio. BMI: Body mass index. HbA1c: glycated hemoglobin HR: heart rate. SBP: systolic blood pressure.

## Discussion

In a large cohort of Brazilian civil servants, we found that a 1 m/s increase in the PWV was associated with a 10% increase in the odds of eGFR <60 ml/min/1.73m^2^, 10% in the odds of ACR ≥30 mg/g and 12% in the odds of presenting CKD, even after adjusting for sociodemographic, behavioral and clinical factors. In addition, by excluding subjects with hypertension and diabetes, we found that these associations remained statistically significant and with slightly greater magnitudes.

Our findings reveal that there is a relation between increased arterial stiffness and renal dysfunction, suggesting that arterial stiffness may contribute to the genesis of CKD, regardless of established CVD risk factors, or that arterial stiffness may be a consequence of CKD and possibly involved in the greater risk of cardiovascular events in individuals with CVD. Moreover, the fact that the magnitude of the associations is slightly greater in individuals with no hypertension and diabetes suggests that the effect of arterial stiffness on worsening of renal function does not require the presence of hypertension or diabetes, which are the main risk factors for CKD and related to greater arterial stiffness [35–37].

Note that the magnitude of the crude association between PWV and eGFR decreased particularly after adjusting for age. Some studies have demonstrated that the number of nephrons, and consequently the area of ​​glomerular filtration, decrease with advancing age, even in the absence of any comorbidities. The greatest decline in the OR for high ACR associated with PWV occurs after adjusting for blood pressure. This may happen because changes in blood pressure levels can damage renal glomeruli, that is, cause endothelial damage, and result in an increased vascular permeability, culminating in proteinuria and loss of glomerular filtration capacity [6,18]. Hypertension and diabetes may weaken the glomerular basal membrane and decrease the number of podocytes [38–40]. It is worth noting that the increase in the PWV may precede the rise in blood pressure and the consequent use of antihypertensive drugs [10] and that, therefore, these two factors can be considered as part of the causal link between increased PWV and renal dysfunction.

The final results observed are consistent with several studies looking into the cross-sectional relation between increased arterial stiffness and abnormal renal function, both in general populations [18,41,42] and in patients with established CKD [19,23]. Increased arterial stiffness is also associated with changes in renal function in other population-based studies [15,20] or selected patients, such as those with diabetes [43,44]. In a population-based cohort study in China with 7154 subjects with a mean age of 54 years, a 1 m/s increase in PWV was able to predict a 15% increase (95%CI: 1.07–1.23) in the odds of presenting proteinuria after 3 years of follow-up [13]. In 2129 subjects of the "Health ABC Study", a 1 m/s increase in the PWV led to a 39% increase in the odds of developing CKD (95%CI: 1.09–1.77) after 9 years of follow-up [15].

Complex pathophysiological mechanisms may explain the associations found in this study. Our results support the hypothesis that greater arterial stiffness generates a high pulsatility flow in the kidneys, which have low impedance and low resistance vascular bed, and are more prone to structural damage to their small arterioles and capillaries in the presence of greater pulsatility [7,10]. Woodard *et al*., 2014, conducted a mediation analysis on the inverse relation between aortic stiffness and eGFR in elders, and found that 34% of this relation is mediated by high pulsatility [45]. Local control of renal blood flow is mediated, in part, by the myogenic tone of resistance vessels, and this self-regulation protects the kidneys from systemic blood pressure oscillations. When the perfusion pressure increases, vessels contract in an attempt to maintain constant blood flow and eGFR which, in turn, increases resistance, limits hyperperfusion of the organ, and may lead to excessive vasoconstriction and structural damage to microvessels [1,5,15]. Thus, poor renal autoregulation seems to play an important role in the pathogenesis of progressive glomerular injury [46,47], which, over time, may lead to increased vascular patency, culminating in proteinuria and loss of glomerular filtration capacity. In addition, the increase of PWV may contribute to renal microvascular endothelial dysfunction [48], which would result in loss of vasodilating capacity due to lower bioavailability of nitric oxide [6].

Although our results corroborate evidences from longitudinal studies of a potential causal role of increased PWV on renal dysfunction, they come from a cross-sectional study, and cannot provide evidence of causality. It is worth mentioning that CKD itself or impaired renal function may accelerate arterial stiffness, i.e. there may be a bidirectional relation between arterial stiffness and renal function [5]. Deterioration of renal function could promote increased arterial stiffness due to the presence of risk factors for CVD or through hemodynamic and metabolic mechanisms, such as hypervolemia secondary to CKD, abnormal calcium and phosphorus metabolism—leading to calcification of vessel walls, the direct effect of uremic toxins, and hyperactivation of the renin-angiotensin system [3,4,7]. In addition, patients with decreased renal function also show signs of systemic inflammation with high levels of cytokines, and studies have shown a relation between chronic inflammation and loss of vascular compliance leading to subclinical arterial stiffness in CKD [12,49]. However, it is worth noting that only 71 subjects in this study (0.52% of the participants) had a eGFR of less than 45 ml/min/1.73m^2^ and the same number had very abnormal *ACR (greater or equal 300 mg/g*), circumstances under which a greater effect of renal function on increasing arterial stiffness is observed.

Other studies found results that diverge from those presented here. Results from the 3^rd^ generation of the Framingham Heart Study showed that higher PWV did not help predict increased eGFR (OR: 1.06; 95%CI: 0.89–1.26), but helped predict progression from normo- to microalbuminuria (OR: 1.19; 95%CI: 1.01–1.40). The authors suggested that this was probably due to the young age of participants (mean age 40 years) [36]. A study with 1717 subjects with eGFR between 59 and 30 mL/min/1.73 m^2^; mean age 73 years, found that arterial stiffness was not statistically associated with eGFR, and only a weakly associated with albuminuria [50]. The inclusion criteria and adjustment factors can also explain differences in results across studies. For example, some studies on PWV and renal dysfunction do not adjust the analysis for HR [17,22], which can influence PWV through the effect of ejected blood volume variations [5].

Regarding the analysis restricted to subjects with no hypertension and diabetes, our findings suggest that there may be a direct and slightly greater effect of increased arterial stiffness in worsening renal function, not mediated by hypertension and/or diabetes. Considering that arterial stiffness contributes to the pathogenesis of hypertension [10], we hypothesize it could also be directly implicated in the genesis of CKD, regardless of hypertension and diabetes. Liu *et al*. (2010), however, identified a much stronger association between arterial stiffness and albuminuria in subjects with diabetes or hypertension compared to those without these conditions [18]. However, in the aforementioned study, the non-hypertension subgroup included individuals with diabetes, just as the non-diabetes subgroup included subjects with hypertension, and the analysis did not exclude subjects with established CVD. In our study we also evaluated the association of PWV with low eGFR and CKD, and included adjustments for important factors, such the use of antihypertensive or antidiabetic drugs, not addressed in the study by Liu *et al*.

Our main limitation lies in the cross-sectional nature of the study, that precludes determining the direction of the observed associations. Also, we did not investigate clinical factors that contribute to PWV increase and that may result from abnormal renal function, such as changes in calcium and phosphorus levels [3]. The indicators of renal function used in this study were measured at a single time, i.e., we did not consider the duration of at least three months of renal abnormalities, as require to define CKD, because this is not feasible in large epidemiological studies such as ours. Finally, our PWV values are likely to be underestimated, and cannot be directly compared with values obtained in other studies because, in ELSA-Brasil, we did not consider the wave traveled distance from suprasternal notch to carotid artery. However, the resulting measurement errors in PWV are likely to be nondifferential, thus, we believe that the observed associations between PWV and renal function are conservative, i.e., biased towards the null.

However, this study is an addition to further evidence of the association between PWV and renal dysfunction, especially by showing that this association persists in subjects without diabetes and hypertension, suggesting that the relation between arterial stiffness and renal dysfunction may not be mediated by their presence. In the near future, the ELSA-Brasil will allow us to estimate the independent contribution of PWV to the incidence of renal dysfunction, as well as its progression. Because the PWV is being measured after a nine-years follow-up, the bidirectional relation between arterial stiffness and renal function may also be studied in more details.

## Supporting information

S1 Table (DOCX)

S2 Table (DOCX)

S3 Table (DOCX)

S4 Table (DOCX)
