# Supplementary material for: Greater aortic stiffness is associated with renal dysfunction in participants of the ELSA-Brasil cohort with and without hypertension and diabetes
Source: PLoS One. 2019 Feb 4;14(2):e0210522. doi: 10.1371/journal.pone.0210522 (PMC6361418; doi:10.1371/journal.pone.0210522)
Supplement: S1 Table — (DOCX) [file pone.0210522.s001.docx]

**[Supporting](https://www.ncbi.nlm.nih.gov/pmc/articles/PMC5849321/) information**

**S1 Table.** Mean and standard deviation of the distribution of Glomerular Filtration Rate (mL/min/1,73m²), according to age groups and sex. Brazilian Longitudinal Study of Adult Health (ELSA – Brasil) 2008-2010.

| **Age groups**  **(years)** | **MEN** | | | **WOMEN** | | |
| --- | --- | --- | --- | --- | --- | --- |
|  | N | Mean | SD | N | Mean | SD |
| **All ages** | 6186 | 84.6 | 14.8 | 7400 | 87 | 15.3 |
| 35-39 | 512 | 94.6 | 13.7 | 571 | 99.7 | 14.2 |
| 40-44 | 966 | 91.4 | 12.8 | 1064 | 94.9 | 13.9 |
| 45-49 | 1322 | 87.9 | 13.2 | 1546 | 90.7 | 13.8 |
| 50-54 | 1153 | 85.1 | 13 | 1420 | 87 | 13.2 |
| 55-59 | 983 | 81.4 | 12.7 | 1295 | 82.6 | 13.4 |
| 60-64 | 635 | 77.2 | 13.4 | 829 | 78.6 | 12.7 |
| 65-69 | 353 | 73.5 | 14.1 | 469 | 75.7 | 13.2 |
| 70-74 | 262 | 66.7 | 12.6 | 206 | 70.4 | 14.1 |
