# Supplementary material for: Greater aortic stiffness is associated with renal dysfunction in participants of the ELSA-Brasil cohort with and without hypertension and diabetes
Source: PLoS One. 2019 Feb 4;14(2):e0210522. doi: 10.1371/journal.pone.0210522 (PMC6361418; doi:10.1371/journal.pone.0210522)
Supplement: S2 Table — (DOCX) [file pone.0210522.s002.docx]

**S2 Table.** Mean and standard deviation (SD) of the distribution of albumin/creatinine ratio (mg/g), according to age groups and sex. Brazilian Longitudinal Study of Adult Health (ELSA – Brasil) 2008-2010.

| **Age groups**  **(years)** | **MEN** | | | **WOMEN** | | |
| --- | --- | --- | --- | --- | --- | --- |
|  | N | Mean | SD | N | Mean | SD |
| **All ages** | 6186 | 18.3 | 126.3 | 7400 | 13.6 | 74.5 |
| 35-39 | 512 | 7.9 | 20.2 | 571 | 9.9 | 23.4 |
| 40-44 | 966 | 10.8 | 72.5 | 1064 | 10.7 | 27.8 |
| 45-49 | 1322 | 11.8 | 86.2 | 1546 | 12.9 | 71.7 |
| 50-54 | 1153 | 17.7 | 83.6 | 1420 | 11.4 | 21 |
| 55-59 | 983 | 16.4 | 92.9 | 1295 | 14.8 | 55.9 |
| 60-64 | 635 | 31.6 | 249.7 | 829 | 16 | 56.7 |
| 65-69 | 353 | 44 | 227.2 | 469 | 25.6 | 228.6 |
| 70-74 | 262 | 41.0 | 182 | 206 | 14.1 | 27.0 |
