# Supplementary material for: Greater aortic stiffness is associated with renal dysfunction in participants of the ELSA-Brasil cohort with and without hypertension and diabetes
Source: PLoS One. 2019 Feb 4;14(2):e0210522. doi: 10.1371/journal.pone.0210522 (PMC6361418; doi:10.1371/journal.pone.0210522)
Supplement: S3 Table — (DOCX) [file pone.0210522.s003.docx]

**S3 Table.** Mean and standard deviation (SD) of Pulse Wave Velocity (m/s) distribution, according to age groups and sex. Brazilian Longitudinal Study of Adult Health (ELSA – Brasil) 2008-2010.

| **Age groups**  **(years)** | **MEN** | | | **WOMEN** | | |
| --- | --- | --- | --- | --- | --- | --- |
|  | N | Mean | SD | N | Mean | SD |
| **All ages** | 6186 | 9.7 | 1.8 | 7400 | 8.9 | 1.7 |
| 35-39 | 512 | 8.6 | 1.1 | 571 | 7.7 | 0.9 |
| 40-44 | 966 | 8.9 | 1.2 | 1064 | 8.1 | 1.2 |
| 45-49 | 1322 | 9.1 | 1.3 | 1546 | 8.5 | 1.2 |
| 50-54 | 1153 | 9.6 | 3.1 | 1420 | 8.9 | 1.4 |
| 55-59 | 983 | 10.1 | 1.8 | 1295 | 9.3 | 1.5 |
| 60-64 | 635 | 10.5 | 2.0 | 829 | 9.8 | 1.9 |
| 65-69 | 353 | 11.2 | 2.2 | 469 | 10.6 | 2.1 |
| 70-74 | 262 | 12 | 2.5 | 206 | 11 | 2.2 |
