# Supplementary material for: Greater aortic stiffness is associated with renal dysfunction in participants of the ELSA-Brasil cohort with and without hypertension and diabetes
Source: PLoS One. 2019 Feb 4;14(2):e0210522. doi: 10.1371/journal.pone.0210522 (PMC6361418; doi:10.1371/journal.pone.0210522)
Supplement: S4 Table — (DOCX) [file pone.0210522.s004.docx]

**S4 Table.** Distribution of Chronic kidney disease, according to age groups and sex. Brazilian Longitudinal Study of Adult Health (ELSA – Brasil) 2008-2010

| **Age groups**  **(years)** | **MEN** | | **WOMEN** | |
| --- | --- | --- | --- | --- |
|  | **n** | **%** | **n** | **%** |
| **All ages** | 562 | 9.09 | 551 | 7.45 |
| 35-39 | 16 | 3.1 | 18 | 3.1 |
| 40-44 | 38 | 3.9 | 39 | 3.6 |
| 45-49 | 63 | 4.7 | 59 | 3.8 |
| 50-54 | 91 | 7.8 | 87 | 6.1 |
| 55-59 | 92 | 9.3 | 123 | 9.5 |
| 60-64 | 87 | 13.7 | 88 | 10.6 |
| 65-69 | 89 | 25.2 | 69 | 14.7 |
| 70-74 | 86 | 32.8 | 68 | 33 |
